# Cannula characteristics and mortality risk in Gluteal Lipofilling: does diameter and tip design matter? A narrative review

**DOI:** 10.1016/j.jpra.2026.08.037

**Published:** 2026-09-02

**Authors:** Cesar Ghadbane, Rateb Saad, Saad Aad, Hagop Sassounian, Carl Karam, Joe Farah, Rawad Hakim, Elie Dagher, Rawad Rachwan

**Affiliations:** a Faculty of Medicine, University of Balamand, El Koura, Lebanon; b Department of Plastic Surgery, Faculty of Medicine, University of Balamand, El Koura, Lebanon; c Department of Plastic Surgery, Notre Dame Des Secours University Hospital, Jbeil, Lebanon

**Keywords:** Gluteal fat grafting, Brazilian butt lift, Cannula, Fat embolism, Lipofilling, Patient safety

## Abstract

Gluteal fat grafting, or Brazilian butt lift, is a rapidly growing aesthetic procedure but remains associated with the highest mortality among cosmetic operations, primarily because of fatal pulmonary fat embolism after injury to the deep gluteal veins. Although safety discussions have focused mainly on injection plane, cannula diameter, tip design, rigidity and port configuration are modifiable technical factors that may influence vascular penetration and intravascular fat delivery. This narrative review synthesised English-language evidence from PubMed/MEDLINE, Embase and the Cochrane Library, supplemented by society advisories, cadaveric studies, biomechanical data, clinical series and forensic reports. Survey and autopsy data link fatal embolism to deep intramuscular injection, downward cannula angulation and cannulas smaller than 4 mm. Cadaveric studies show that subcutaneous vessels are approximately 1 mm in diameter, whereas the superior and inferior gluteal veins measure about 7.6 mm and 13.7 mm, respectively, supporting the rationale for large-bore blunt cannulas only when maintained in the subcutaneous plane. Dynamic studies demonstrate that subfascial or intramuscular fat can migrate into deep compartments and enter injured veins, whereas subcutaneous fat does not. Current consensus recommendations therefore support subcutaneous-only injection using a large-bore (≥4 mm), blunt, rigid cannula with continuous motion, low-pressure delivery and real-time ultrasound confirmation. However, evidence remains indirect, with no controlled studies isolating cannula geometry as an independent determinant of mortality. Standardised reporting and prospective registries are needed.

## Introduction

Gluteal augmentation by autologous fat transfer also known as Brazilian Butt Lift (BBL), has become one of the fastest-growing procedures in aesthetic surgery, with hundreds of thousands performed annually and sustained growth over the past decade.^1^^,^^2^ Its popularity reflects an evolution in body-contouring ideals and the appeal of using a patient’s own adipose tissue to enhance gluteal projection and shape. However, gluteal fat grafting is associated with the highest mortality of any aesthetic surgical procedure, with early estimates of fatal events on the order of 1 in 3000.^1^^,^^3^

The mechanism of death is now well characterised. Fatal outcomes are overwhelmingly attributable to pulmonary fat embolism caused by the entry of grafted fat into the low-pressure gluteal venous system leading to embolisation in the pulmonary circulation and mechanical obstruction and acute cardiopulmonary collapse.^3^, ^4^, ^5^, ^6^ Forensic series consistently identify in patients who have died after the procedure a zone of injury around the large deep gluteal veinswith grafted fat in or around these vessels.^4^^,^^5^

In response, attention has focused on the technical determinants of vascular injury. Debate has centred on the injection plane: subcutaneous versus intramuscular or submuscular, but the cannula trajectory, the recipient-site injection pressure and the physical characteristics of the injection cannula itself have all been implicated.^7^, ^8^, ^9^, ^10^ The injection cannula is the instrument that interfaces directly with the recipient tissues and vessels. Its diameter, tip geometry, number of ports, rigidity and length plausibly modulate the ease with which a vessel wall can be penetrated and the pressure at which fat is delivered. Yet, relative to the plane debate, the contribution of cannula characteristics has received limited systematic attention, and clinical reports seldom specify the instrumentation used.^1^^,^^8^

The aim of this narrative review is to examine the available evidence on how cannula diameter and tip design relate to vascular injury and mortality in gluteal lipofilling, and to integrate these data into the current framework of consensus safety recommendations.

## Methods

This article is a narrative review. The English-language literature was searched in PubMed/MEDLINE, Embase and the Cochrane Library from inception to 2025 using combinations of the terms “gluteal fat grafting”, “Brazilian Butt Lift”, “buttock augmentation”, “lipofilling”, “fat embolism”, “pulmonary fat embolism”, “cannula”, “cannula diameter”, “blunt cannula”, “injection pressure”, “gluteal vein” and “ultrasound-guided”. Reference lists of retrieved articles, relevant textbook chapters and the published safety advisories of major plastic surgery organisations like the Aesthetic Surgery Education and Research Foundation (ASERF), the Multi-Society Gluteal Fat Grafting Task Force, The Aesthetic Society, the American Society of Plastic Surgeons and the International Society of Aesthetic Plastic Surgery, were hand-searched for additional sources. Anatomical and cadaveric studies, biomechanical investigations, clinical case series, survey-based epidemiological studies, forensic and autopsy reports, and consensus guidelines were considered. Priority was given to studies that reported cannula specifications, gluteal vascular calibre, fat-migration behaviour, injection pressures, or procedure-related morbidity and mortality. A qualitative synthesis was undertaken rather than a formal systematic review or meta-analysis due to the heterogeneity of study designs and the scarcity of controlled comparative data. This approach is acknowledged as a source of selection bias. The key studies and consensus statements included in the narrative synthesis are summarized in Table 1.

**Table 1:** Selected key studies and statements on mortality and safety in gluteal fat grafting, in chronological order.

Study (first author, year)	Design	Key finding relevant to safety / cannula
Cárdenas-Camarena, 20,15^3^	Multinational survey + autopsy registry (Mexico/Colombia)	22 pulmonary fat-embolism deaths, all associated with intramuscular lipoinjection; first major alarm.
Mofid (ASERF Task Force), 20,17^1^	International surgeon survey + forensic data	Highest mortality of any aesthetic procedure (∼1/3000); deaths associated with deep-muscle injection, downward cannula angulation and cannulas <4 mm.
Del Vecchio, 20,18^9^	Dynamic cadaveric study	Intramuscular fat migrates into the submuscular space; subcutaneous fat does not.
Bayter-Marin, 20,18^5^	Clinical records + autopsy review (16 patients)	Characterised fatal fat embolism; injury centred on the deep gluteal veins.
Wall, 201,9^10^	Dynamic cadaveric study with manometry	Gluteus maximus fascia acts as a barrier; subfascial injection promotes deep migration; proposed subcutaneous-only standard.
Ordenana, 202,0^11^	Latex-cast cadaveric calibre study	Subcutaneous vessels ∼1 mm; superior/inferior gluteal veins ≈7.6/13.7 mm; deepest and most medial planes house the largest vessels.
Rios & Gupta, 202,0^21^	Pooled survey analysis	Mortality and embolism reduced after adoption of ASERF/ASAPS/ISAPS recommendations.
Del Vecchio & Kenkel (Practice Advisory), 20,22^8^	Consensus advisory	Subcutaneous-only injection; stiff cannula; avoid Luer-lock; address operative tempo and fatigue.
Pazmiño & Garcia, 20,23^4^	Regional mortality analysis (South Florida)	Surgeons believed injection was subcutaneous yet embolism deaths occurred; supports real-time ultrasound and case-volume limits.
Pazmiño & Del Vecchio (SIME), 202,3^19^	Ultrasound-guided technique description	Static, ultrasound-confirmed subcutaneous injection removes cannula malposition.
Finkelstein, 20,24^2^	Surgeon survey (USA)	BBL remains the deadliest aesthetic procedure; uptake of ultrasound still variable.

ASERF, Aesthetic Surgery Education and Research Foundation; ASAPS, American Society for Aesthetic Plastic Surgery; ISAPS, International Society of Aesthetic Plastic Surgery; BBL, Brazilian Butt Lift; SIME, static injection, migration and equalization.

### Overview of fat injection in gluteal lipofilling

Gluteal fat grafting follows the general three-stage workflow of all autologous fat transfer: harvest, processing and reinjection. Adipose tissue is commonly aspirated by liposuction the abdomen, flanks, back and thighs. It is then processed by decantation, filtration, centrifugation or washing to separate viable adipocytes from blood, oil and infranatant fluid.^7^^,^^8^ Finally it is reinjected into the gluteal region to augment volume and refine contour.^7^^,^^8^

Reinjection technique is governed by several principles intended to maximise graft survival and aesthetic outcome. Fat is classically delivered in small aliquots distributed across multiple tissue tunnels to maximise the graft-to-recipient interface and revascularization.^7^^,^^8^ A retrograde technique (depositing fat as the cannula is withdrawn) and a fanning motion from each access incision is used to build volume in layers.^7^^,^^8^ Continuous cannula motion during deposition is emphasised to distribute fat evenly and to avoid sustained delivery of a fat bolus into a single point that may correspond to a vessel lumen.^7^^,^^8^

Instrumentation varies. Fat may be delivered manually from syringes or by mechanical means such as peristaltic (“roller”) pumps or expansion-vibration systems; powered delivery reduces operator hand fatigue during the large-volume transfers typical of gluteal augmentation.^8^ Injection cannulas used for the buttock are generally longer and of larger bore than those used for facial or breast grafting thus, larger volumes and deep recipient sites are involved.^1^^,^^7^^,^^8^ External diameters of 3–5 mm and lengths of approximately 15–25 cm are commonly described, and society guidance has converged on a minimum diameter and on rigid rather than flexible cannulas.^1^^,^^7^^,^^8^

The recipient plane has been the central safety question. Two principal planes have historically been used: the subcutaneous fat plane (superficial to the gluteus maximus fascia) and the intramuscular plane (within, or deep to, the gluteus maximus). Historically, intramuscular and even submuscular placement was advocated by some to achieve greater projection and graft retention.^3^ However, the recognition that the large thin-walled gluteal veins lie within and deep to the muscle and that these vessels are the conduit for fatal embolism, has driven a decisive shift toward subcutaneous-only injection in contemporary safety recommendations.^7^, ^8^, ^9^, ^10^ This anatomical and technical reorientation provides the context for considering how the cannula itself influences the risk of vascular injury.

### Pathophysiology of fatal fat embolism

The lethal event in gluteal lipofilling is the entry of grafted fat into the venous circulation in sufficient volume to obstruct the pulmonary vasculature. Two broad mechanisms have been proposed, and they are not mutually exclusive: direct intravascular injection through a breached vein wall, and the mobilisation of extravascular fat into veins that have been torn or opened during cannula passage.^3^^,^^5^^,^^9^^,^^10^

Direct vascular injury occurs when the cannula tip penetrates or lacerates a gluteal vein and fat is delivered directly into the lumen under pressure. The deep gluteal veins offer little resistance to the ingress of fat when breached due to their thin walls and valveless channels and large-calibre, particularly when injection is performed with force.^3^^,^^11^ Intramuscular venous sinuses and the rich perforating network of the gluteus maximus provide additional portals of entry.^9^^,^^12^ A second, complementary mechanism is suggested by dynamic cadaveric work. Fat deposited beneath the gluteus maximus fascia can migrate through the muscle into the submuscular space. So if a vein has been injured, the fat may be drawn into the circulation.^9^^,^^10^ Fat placed in the subcutaneous plane, by contrast, does not migrate into the deep compartments.^9^^,^^10^ High recipient-site pressure is common in both mechanisms, as elevated intramuscular pressure may both promote fat migration and drive fat across a vessel breach.^10^

The relevant venous anatomy is now well defined. The gluteal region is supplied and drained by the superior and inferior gluteal vessels.^11^^,^^13^ These vessels exit the pelvis through the greater sciatic foramen above and below the piriformis, respectively, and ramify within and deep to the gluteus maximus.^11^^,^^13^ Cadaveric calibre studies are striking: the superior and inferior gluteal veins have been measured at approximately 7.6 mm and 13.7 mm in diameter, respectively.^11^ Their named branches measure 3–4 mm, whereas the vessels of the subcutaneous plane are on the order of 1 mm.^11^ The deeper and more medial the dissection, the larger and more numerous the vessels, precisely the territory of the most dangerous injections.^11^ (Table 2)

**Table 2:** Approximate calibre of gluteal vessels by anatomical plane and its relationship to a typical injection-cannula diameter (synthesised from Ordenana et al.^11^).

Anatomical plane / vessel	Approx. diameter (mm)	Relationship to a blunt ≥4 mm cannula
Subcutaneous artery	≈ 0.9	Much smaller than cannula — tip tends to deflect off, not enter.
Subcutaneous vein	≈ 1.05	Much smaller than cannula — low risk of intraluminal entry.
Intramuscular (gluteus maximus) vein	≈ 1.3	Smaller than cannula.
Inferior gluteal branch (artery / vein)	≈ 2.2 / 3.5	Approaching cannula calibre.
Superior gluteal branch (artery / vein)	≈ 1.8 / 3.85	Approaching cannula calibre.
Superior gluteal vein (trunk)	≈ 7.6	Larger than cannula — can admit the tip.
Inferior gluteal vein (trunk)	≈ 13.7	Much larger than cannula — readily admits the tip.

Values are approximate means from a latex-cast cadaveric study^11^ and illustrate the calibre gradient that underlies the rationale for large-bore, subcutaneous-only injection. They are not thresholds for clinical decision-making.

Once fat enters the venous return, it travels to the right heart and pulmonary arteries. Embolic obstruction of the pulmonary vasculature produces acute right ventricular strain and failure, ventilation–perfusion mismatch and hypoxemia.^3^, ^4^, ^5^^,^^12^ When the embolic burden is large (macroscopic fat embolism), it will lead to rapid intraoperative or early postoperative cardiovascular collapse and death.^3^, ^4^, ^5^^,^^12^This macroscopic syndrome is distinct from classic post-traumatic fat embolism syndrome which typically follows long-bone fractures. Fat embolism syndrome evolves over 24–72 h through a biochemical cascade and presents with the triad of respiratory insufficiency, neurological dysfunction and petechiae.^5^^,^^12^ In gluteal lipofilling, although a microscopic delayed picture can occur, the deaths that have made the procedure an outlier are predominantly due to acute macroscopic embolism occurring during or immediately after injection.^4^^,^^5^^,^^12^ Survival after massive gluteal fat embolism is rare and has only occasionally been reported.^14^

### Cannula characteristics in fat grafting

The injection cannula can be characterised by four interrelated physical attributes: diameter, tip geometry, port configuration, and rigidity and length. Each characteristic has been argued to bear on safety and on graft handling. (Table 3)

**Table 3:** Cannula characteristics and their hypothesised influence on safety and graft handling.

Characteristic	Options described	Hypothesised safety rationale	Principal caveat
Diameter	3, 4 or 5 mm	≥4 mm exceeds subcutaneous vessel calibre and lowers injection pressure (Poiseuille); sub-4 mm use associated with mortality in survey data.^1^^,^^11^^,^^15^	Confounded with injection plane; no controlled comparison.
Tip geometry	Blunt, single-hole blunt, spatulated, Mercedes (multi-hole)	Blunt tips push vessels aside rather than incise them.^7^^,^^16^	Direct comparative data lacking.
Port configuration	Single distal port vs multiple side ports	Multiple ports diffuse delivery and may lower per-orifice pressure and volume.	Effect on embolic risk untested; single port aids directional precision.
Rigidity	Rigid vs flexible	Rigid cannulas follow a predictable plane and transmit feedback, reducing inadvertent deep deflection.^7^^,^^8^	—
Length	≈ 15–25 cm	Reach deep recipient sites from few access incisions.	Long lever arm magnifies angular error at the tip.^7^

**Diameter.** Cannulas for gluteal injection are commonly 3, 4 or 5 mm in external diameter. Diameter influences three variables of interest. First, it determines the relationship between the instrument and the vessels it may encounter. A cannula whose external diameter exceeds the calibre of a vessel is mechanically less able to enter that vessel’s lumen, whereas a cannula narrower than the vessel can pass within it. Given that subcutaneous gluteal vessels measure ∼1 mm and the deep gluteal veins 8–14 mm,^11^ a blunt cannula of ≥4 mm is substantially wider than the small subcutaneous vessels it meets in the safe plane but remains far narrower than the deep veins encountered with intramuscular passage. Second, diameter governs injection pressure for a given flow. By the Hagen–Poiseuille relationship, the pressure required to drive a viscous material through a tube rises steeply as the radius falls (in inverse proportion to the fourth power of the radius). So narrower cannulas demand higher pressures to deliver fat at a given rate which increases the force available to drive fat across any vascular breach.^8^^,^^15^ Third, diameter constrains the size of the fat parcels that can pass without obstruction and shear, with implications for graft viability.^15^

**Tip design.** Several tip geometries are in use. Those include blunt (closed, rounded) tips, single-hole blunt cannulas, spatulated tips, and multi-hole configurations such as the “Mercedes” tip. The central safety rationale for a blunt tip is that it tends to push vessels and other mobile structures aside rather than transect them such as in sharp or bevelled tip that can incise a vessel wall.^7^^,^^16^ Cadaveric and consensus work consistently favours a blunt-tipped cannula for gluteal injection to minimise inadvertent venotomy and tissue dissection.^7^^,^^16^ Tip geometry also shapes the dispersal pattern of delivered fat and the directionality of flow.

**Port configuration.** Cannulas may have a single distal port or multiple side ports. Multi-port cannulas distribute fat across several openings which allows the lowering of pressure and volume delivered through any single orifice and producing a more diffuse deposition. Whereas single-port cannulas concentrate delivery at one point affording more precision, but if that point lies within a vessel, the entire bolus will be delivered intravascularly. The implications of port number for embolic risk have not been directly tested, and the trade-off between diffuse low-pressure delivery and predictable directional control remains a matter of surgical judgement.

**Rigidity and length.** Buttock injection cannulas are relatively long in order to reach the recipient sites from a limited number of access incisions. A rigid cannula transmits the surgeon’s proprioceptive feedback more faithfully and follows a more predictable and controlled trajectory which helps keeping the tip in the intended plane. A flexible cannula on the other hand, may deflect along tissue planes of least resistance and can be unintentionally directed into the muscle. This is a concern that underlies the recommendation to use stiff cannulas and to avoid a Luer-lock interface that could allow the cannula to disengage from the intended path.^7^^,^^8^ Increasing Length by increasing the lever arm, can amplify small angular errors at the hub into large positional errors at the tip.^7^ Together, these attributes frame the specific evidence linking cannula design to vascular injury, considered next.

### Evidence linking cannula design to safety

The evidence relating cannula characteristics to safety in gluteal lipofilling is drawn from four sources: survey and forensic epidemiology, anatomical calibre mapping, dynamic migration studies, and fluid-mechanical reasoning. While none of these sources provide a controlled comparison of cannulas, if taken as a whole they provide a coherent picture even if indirect.

**Survey and forensic epidemiology.** ASERF Task Force survey of gluteal fat grafting, which gathered both surgeon-reported complications and forensic records, offers the most influential data point.^1^ Three technical factors were identified for avoindance among the factors associated with fatal and nonfatal embolism: injection into the deep muscle, downward angulation of the cannula and the use of cannuals smaller than 4 mm.^1^ This is the principal published evidence directly implicating cannula diameter in mortality, and it underlies the subsequent recommendation for a minimum 4 mm bore. Forensic series confirm the anatomical locus of injury: autopsy reviews of gluteal-lipoinjection deaths consistently describe macroscopic fat within or adjacent to the deep gluteal veins and a surrounding zone of tissue injury, indicating that a breach of these large deep vessels is the proximate lethal event.^4^^,^^5^^,^^12^^,^^17^ (Table 4).

**Table 4:** Summary of consensus recommendations on cannula use and injection technique (ASERF / Multi-Society Task Force / Practice Advisory).

Recommendation	Rationale
Inject into the subcutaneous plane only (above the gluteus maximus fascia).	Deep veins lie within and deep to muscle; subcutaneous fat does not migrate to the deep compartment.^9^^,^^10^
Use a large-bore cannula (≥4 mm).	Exceeds subcutaneous vessel calibre and lowers injection pressure.^1^^,^^11^
Use a rigid, blunt-tipped cannula.	Predictable trajectory; pushes vessels aside rather than incising them.^7^^,^^16^
Avoid downward (pelvic-directed) cannula angulation.	Reduces aim toward the deep gluteal vessels.^1^
Maintain continuous cannula motion; avoid stationary bolus delivery.	Prevents sustained intravascular delivery at a single point.^7^^,^^8^
Maintain constant awareness of the cannula-tip location.	The tip may stray from the intended plane unrecognised.^4^^,^^7^
Avoid forceful, high-pressure injection; consider low-pressure delivery.	High pressure promotes migration and intravascular delivery.^8^^,^^10^
Use real-time ultrasound to confirm subcutaneous tip position.	Blind technique cannot confirm plane; deaths occur despite intended subcutaneous injection.^4^^,^^18^^,^^19^
Limit grafted volume and operative tempo; mitigate fatigue and distraction.	Fatigue and distraction are linked to inadvertent deep injection.^8^

**Anatomical calibre mapping.** Cadaveric calibre mapping provides the anatomical basis for this interaction, showing substantially smaller vessels superficially and larger gluteal veins in deeper planes.^11^

**Dynamic migration studies.** Dynamic cadaveric studies further support the importance of injection plane, demonstrating migration into deeper compartments after subfascial injection but not after subcutaneous injection.^9^^,^^10^

**Fluid-mechanical considerations.** The pressure required to deliver fat is a function of cannula geometry and fat rheology. Narrow cannulas require significantly higher driving pressures to maintain a given delivery rate because resistance to flow through a cannula scales inversely with the fourth power of its radius.^8^^,^^15^ This both increases the force that could propel fat across a vascular breach and tends to fragment fat parcels.^8^^,^^15^ Ex vivo work on liposuction and fat handling supports the more general principle that mechanical forces during fat mobilisation can liberate free fat capable of embolisation.^15^ Larger-bore cannulas, conversely, permit low-pressure delivery of intact fat parcels, consistent with the clinical preference for large cannulas combined with controlled, motion-based, low-pressure deposition.^7^^,^^8^ Powered low-pressure delivery systems and the avoidance of forceful syringe injection follow the same logic.^8^

**Synthesis.** Taken together, the available evidence suggests that cannula characteristics influence safety primarily through their interaction with injection plane, vessel calibre and delivery pressure. However, the independent contribution of cannula geometry remains uncertain because available evidence is largely anatomical, cadaveric, biomechanical or observational.^1^^,^^8^

### Current surgical safety recommendations

Current consensus recommendations integrate cannula characteristics within a broader safety strategy that includes subcutaneous-only injection, large-bore (≥4 mm), rigid and blunt cannulas, continuous motion, avoidance of downward angulation, and maintenance of cannula-tip awareness.^1^^,^^7^^,^^8^ These measures are intended to reduce the likelihood of deep-plane vascular injury rather than to provide independent protection from embolism.

A major recent development is the incorporation of real-time intraoperative ultrasound to confirm that the cannula tip remains in the subcutaneous plane. The weakness of the blind technique: a surgeon may believe injection is subcutaneous while the tip has entered the muscle, is addressed by ultrasound.^4^ Favorable safety profiles have been reported in both prospective and retrospective series of ultrasound-guided, subcutaneous-only grafting.^18^^,^ ^19^ Methods like static injection with ultrasound confirmation (injecting only when the stationary tip is visualised above the deep fascia) have been described to eliminate cannula malposition from the procedure.^18^^,^ ^19^ Florida Board of Medicine restricted intramuscular injection and subsequently issued emergency rules limiting surgeons to three gluteal fat grafting procedures per day and mandating ultrasound guidance of cannula position during injection.^4^ The same concept of real-time tip localization is extended to the instrument itself with adjuncts under development, such as impedance-sensing "smart" cannulas that signal when the tip leaves the subcutaneous plane.^20^ The unifying objective across all of these measures is to keep an appropriately designed cannula in the correct plane and to deliver fat at a low pressure away from the large deep veins.

### Limitations of the existing evidence

The evidence base relating cannula characteristics to safety has important limitations. First, there are no randomised controlled trials and no comparative clinical studies evaluating cannula diameter, tip design, port configuration or rigidity against embolic or mortality endpoints. The rarity of fatal events and the obvious ethical barriers make such trials impractical, so the field relies on lower tiers of evidence.^1^^,^^8^ Second, much of the mechanistic support derives from cadaveric and biomechanical models that, however elegant, cannot fully reproduce the perfusion, venous pressure, tissue tone and physiological responses of a living patient, and typically use proxy materials rather than processed human fat.^9^^,^^10^ Third, the clinical and forensic literature rarely include details about the cannula such as diameter, tip type, ports, rigidity and length.^1^^,^^8^ Thus, instrument’s contribution cannot be separated from other variables in real-world deaths.^1^^,^^8^ Fourth, surgical technique is highly heterogeneous across studies and surgeons. Variations in plane, pressure, volume, delivery method and anaesthesia also occur leading to producing substantial confounding.^1^ Fifth, the principal epidemiological signal implicating sub-4 mm cannulas originates from a voluntary survey subject to recall and reporting bias, and denominators for procedure volume are uncertain, limiting the precision of mortality estimates.^1^^,^^21^ Finally, the specific contribution of cannula selection cannot be quantified since the apparent improvement in mortality after dissemination of the guidelines reflects the combined adoption of multiple measures.^19^^,^^21^ These constraints mean that the recommendation to use large, blunt, rigid cannulas, although biologically reasoned and widely endorsed, rests on indirect evidence and consensus rather than on direct demonstration of an independent mortality benefit.

### Future directions

The evidence could be strengthened and improved in a number of ways. The current lack of denominator data would be addressed by prospective, multi-institutional registries that collect standardized intraoperative data such as: cannula diameter, tip geometry, port number, rigidity and length, along with plane, delivery method, pressure, volume, and outcomes.^8^^,^ ^21^ This would enable the independent contribution of instrumentation to be evaluated at scale.^8^^,^^21^ Standardized reporting of surgical technique and instrumentation in published series, ideally through an agreed minimum dataset, is a necessary precondition for such analyses and would itself improve the interpretability of the literature.^1^^,^^8^ Controlled biomechanical and benchtop studies could directly compare the force required to penetrate vessel-mimicking and cadaveric vessels with cannulas of differing diameter and tip design. This could quantify intraluminal delivery pressures across cannula geometries and delivery systems, thereby testing the fluid-mechanical assumptions that underpin current recommendations.^11^^,^^15^ To help define how reliable the cannula tip is to be maintained in the subcutaneous plane, a continued evaluation of real-time imaging is necessary.^18^^,^^19^ It is necessary to conduct a prospective clinical evaluation of instrument innovation, such as impedance-sensing and flow-limiting "smart" cannulas and powered low-pressure delivery systems. This will determine whether engineering the cannula to stay in or signal departure from the proper plane results in quantifiable safety improvements.^20^ Finally, integrating cannula-design considerations explicitly into structured training and simulation may help to ensure that the theoretical safety advantages of appropriate cannula selection are realized in practice.^8^^,^^19^ Collectively, these efforts would move the field from plausibility and consensus toward direct, quantitative evidence.

## Discussion

Gluteal lipofilling occupies an uncomfortable position in aesthetic surgery: a procedure of enormous and growing demand that remains the most lethal cosmetic operation, with deaths concentrated in a single, largely preventable mechanism.^1^^,^^2^^,^^4^ This review has examined the cannula (the instrument at the point of contact between surgeon and vasculature) as a modifiable contributor to that mechanism. The synthesis points to a consistent conclusion that cannula characteristics matter. These characteristics matter chiefly through their interaction with gluteal vascular anatomy, injection plane and injection pressure rather than as an independent lever.

For the practicing surgeon, this reframing has a clear implication. The instrument cannot substitute for the plane. So a large, blunt, rigid cannula used carelessly in the deep muscle is not protective, whereas the same cannula used in the subcutaneous plane with continuous motion, low pressure and ultrasound confirmation embodies the current standard of care.^8^^,^^18^^,^^19^ The regular finding that skilled and high-volume surgeons report few or no fatalities indicates that technique and discipline, of which cannula selection is one component, are crucial.^1^^,^^7^

The principal gap is the absence of direct and controlled evidence isolating cannula geometry.^1^^,^^8^ As well as the near-universal failure to report instrumentation in clinical series.^1^^,^^8^ Until registries and standardised reporting allow the instrument’s independent effect to be quantified, the recommendation to use large, blunt, rigid cannulas must be understood as a biologically sound, broadly endorsed, but indirectly supported component of a multifactorial safety strategy. The most promising near-term gains are likely to come not from the cannula alone but from coupling appropriate cannula design with real-time confirmation that it remains in the safe plane.^18^^,^^19^^,^^20^

## Conclusion

Mortality in gluteal lipofilling is driven almost entirely by pulmonary fat embolism. The proximate event is the entry of grafted fat into the large, deep gluteal veins. Within this framework, the physical characteristics of the injection cannula (diameter, tip design, port configuration and rigidity) are biologically plausible and modifiable contributors to safety. The available anatomical, cadaveric, biomechanical and epidemiological evidence supports the use of large-bore (≥4 mm), blunt, rigid cannulas, employed in the subcutaneous plane with continuous motion and low delivery pressure, as a means of reducing inadvertent vascular penetration and high-pressure intravascular fat delivery. However, this evidence is indirect and confounded by injection plane. Also no study isolates cannula geometry as an independent determinant of mortality. Optimisation of cannula design, coupled with real-time confirmation of subcutaneous placement and standardised reporting, represents a tractable opportunity to improve the safety of one of aesthetic surgery’s most dangerous procedures.

## Funding

This research project received no funding.

## Ethical approval

Not Applicable.

## Artificial intelligence use statement

During the preparation of this work the authors used ChatGPT (OpenAI) in order to assist with language editing and structural refinement of the text. After using this tool, the authors reviewed and edited the content as needed and take full responsibility for the content of the publication. No AI tools were used for data generation, analysis, or interpretation.

## Declaration of competing interest

Authors have nothing to declare.
